# Supplementary material for: Minimal tool set for a prokaryotic circadian clock
Source: BMC Evol Biol. 2017 Jul 21;17:169. doi: 10.1186/s12862-017-0999-7 (PMC5520375; doi:10.1186/s12862-017-0999-7)
Supplement: Supplementary file 1 — Supplement 1. This file contains all supplementary figures and tables, except of Table S5. (PDF 9260 kb) [file 12862_2017_999_MOESM1_ESM.pdf]

Minimal Tool Set for a Prokaryotic Circadian Clock
Supplemental Material

Nicolas M Schmelling[1], Robert Lehmann[2], Paushali Chaudhury[3], Christian Beck[2], Sonja V Albers[3], Ilka M Axmann*[1], and Anika Wiegard[1]

[1]Institute for Synthetic Microbiology, Cluster of Excellence on Plant Sciences (CEPLAS), Heinrich Heine University Duesseldorf, Universitaetsstr. 1, 40225 Duesseldorf, Germany
[2]Institute for Theoretical Biology, Humboldt University Berlin, Invalidenstr. 43, 10115, Berlin, Germany
[3]Molecular Biology of Archaea, University of Freiburg, Institute of Biology II, Schaenzlestr.1, 79104 Freiburg, Germany

June 6, 2017

*Ilka.Axmann@hhu.de

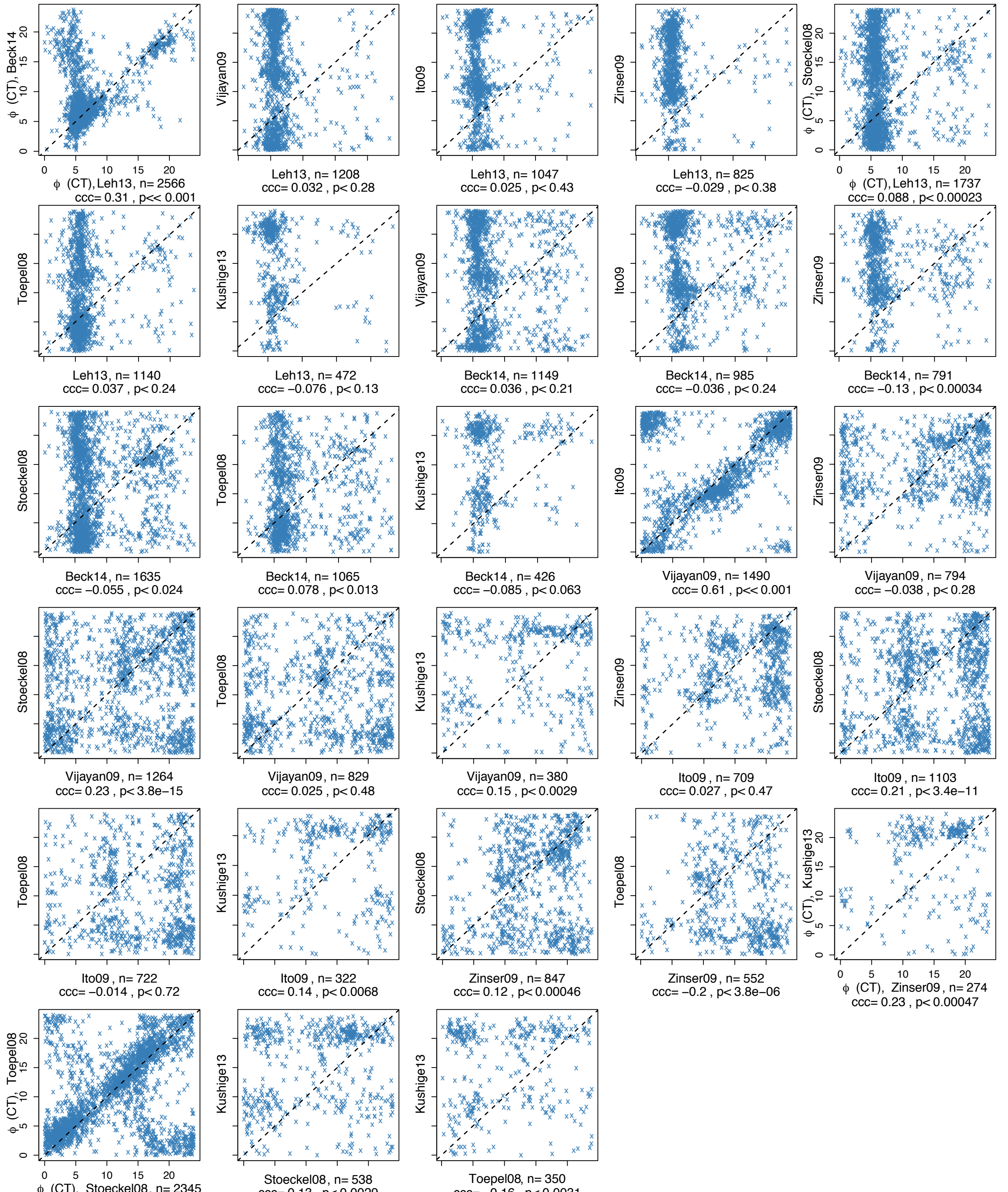

Figure S1: **Expression phase similarity across cyanobacterial strains by pairwise comparison between available datasets.** All possible pairwise combinations of available circadian datasets are compared with respect to peak expression phase, considering only genes, which oscillate significantly (fdr < 0.05) in both datasets. While phases are compared directly for same-strain combinations, gene pairs across different strains are derived via homology prediction. The respective dataset is shown on each axis, the count of homologous genes significantly oscillating is provided with the x-axis label, together with the circular correlation coefficient ρccc and the resulting p-value.

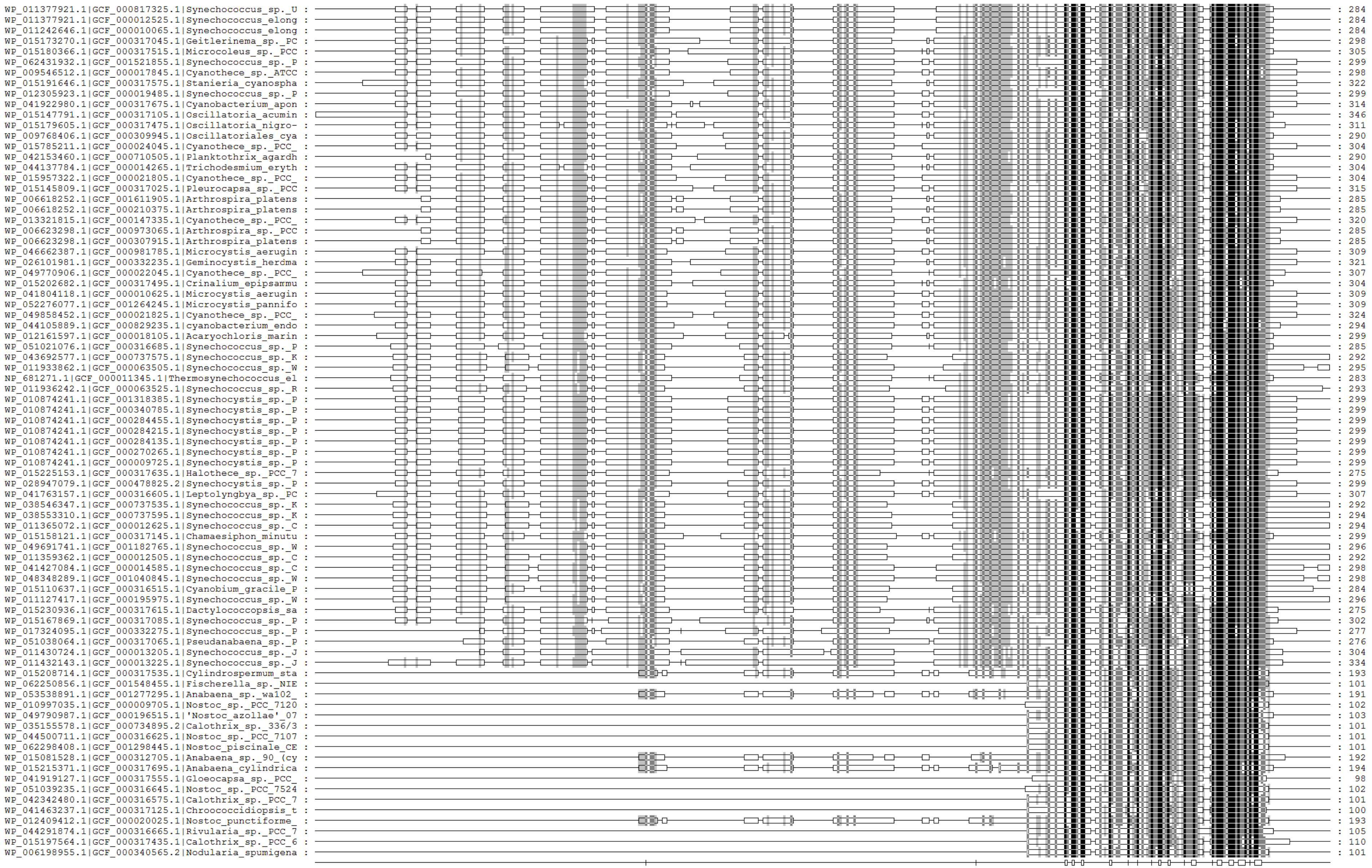

Figure S2: **Multiple sequence alignment of KaiA.** Conserved regions are highlight: ≤100% (black), ≤80% (grey), ≤60% (light grey), ≤40% (white).

Table S1: **Proteins of the circadian clock used as queries for the reciprocal BLAST analysis.** The association of KaiB2/B3 and KaiC2/C3 varies between the Cyanobase Database and [1]

Organism Name	Function	Protein Name	Gene ID
Synechococcus elongatus PCC 7942	Core	KaiA	Synpcc7942_1218
		KaiB	Synpcc7942_1217
		KaiC	Synpcc7942_1216
	Input	Pex	Synpcc7942_0677
		LdpA	Synpcc7942_0624
		NhtA	Synpcc7942_2160
		PrkE	Synpcc7942_0600
		IrcA	Synpcc7942_2383
		CdpA	Synpcc7942_1604
	Input/Output	CikA	Synpcc7942_0644
	Output	SasA	Synpcc7942_2114
		LabA	Synpcc7942_1891
		LalA	Synpcc7942_1143
		Crm	Synpcc7942_0096
		RpaA	Synpcc7942_0095
		RpaB	Synpcc7942_1453
		CpmA	Synpcc7942_1168
Synechocystis sp. PCC 6803	Core	KaiB1	slr0757
		KaiC1	slr0758
		KaiB2	sll1596
		KaiC2	sll1595
		KaiB3	sll0486
		KaiC3	slr1942

Table S2: **Distribution of *Synechococcus* 7942 based circadian clock proteins in the four main groups.** The percentage indicates the coverage of the orthologs per group.

Group	Core Clock	Input Pathway	Output Pathway
Cyanobacteria	KaiA (94.29%), KaiB (94.29%), KaiC (100%)	Pex (62.86%), LdpA (97.14%), CikA (94.29%), NhtA (48.57%), PrkE (85.71%), IrcA (97.14%), CdpA (74.29%)	CikA (94.29%), SasA (100%), LabA (88.57%), LalA (91.43%), Crm (45.71%), RpaA (97.14%), RpaB (100%), CpmA (97.14%)
Proteobacteria	KaiB (28.13%), KaiC (100%)	CikA (56.25%), NhtA (26.56%), PrkE (51.56%)	CikA (56.25%), SasA (32.81%), LabA (4.69%), LalA (39.06%), Crm (20.31%), RpaA (7.81%), RpaB (48.44%), CpmA (21.86%)
Archaea	KaiB (6.06%), KaiC (100%)	CikA (12.12%), NhtA (22.73%), PrkE (13.64%)	CikA (12.12%), SasA (3.03%), LabA (1.51%), LalA (33.33%), Crm (7.58%), CpmA (63.64%)
Other	KaiB (31.43%), KaiC (100%)	CikA (25.71%), NhtA (17.14%), PrkE (71.43%), IrcA (54.29%)	CikA (25.71%), SasA (31.43%), LalA (5.71%), Crm (2.86%), RpaA (11.43%), RpaB (48.57%), CpmA (51.43%)

Table S3: **A collection of circadian and diurnal expression datasets in the cyanobacterial clade.** Datasets with assigned abbreviation were used to determine the core oscillatory genome. Information is provided about the ability to fix nitrogen, the habitat (freshwater F, saltwater S), the total number of genes, the publication reference, the absolute and relative number of diurnally expressed genes reported in the original publications, the applied light and sampling schema, the experimental culture conditions, and the methods for microarray normalisation and oscillating gene detection. References to datasets employed in the following comparison are shown bold.

Strain	Strain Abbrev.	N_2 Fixation	Habitat	Total Genes	Ref.	Dataset Abbrev.	Diurnal Genes	Light Conditions	Culture Conditions	Normalization / Oscillation Detection
Prochlorcoccus marinus MED4	ProMED4	-	M	1766	[2]	zinser09	1403 (79%)	LD (14:10, T_{samp} 2h)	Pro99 Medium, stirred, 24°C, Batch	RMA, Fourier Analysis
Synechocystis sp. PCC 6803	Syn6803	-	F	3628	[3]	leh13	1133 (31%)	LD (12:12, T_{samp} irreg.)	BG11 Medium, air bubbling, 30°C, Batch	LOS, Fourier Analysis
					[4]	beck14	(27%)	LD (12:12, T_{samp} 2h)	BG11 Medium, air bubbling, 30°C, Batch	LOS, Fourier Analysis
					[5]	-	1349 (37%)	LD (14:10, T_{samp} 2h)	BG11 Medium, stirred, 3% CO_2air bubbling, 27°C, Turbidostat	Standorf Microarray Database standard, ANOVA and correlation with light
					[6]	-	237 (9%)	LL (T_{samp} 4h)	BG11 Medium, air bubbling, stirred, 30°C, Batch with manual dilution	LOWESS, modified Cosiner
Synechococcus elongatus PCC 7942	Syc7942	-	F	2719	[7]	vijayan09	1748 (64%)	LL (T_{samp} 4h)	BG11 Medium, 1% air CO_2 bubbling, 30°C, Continuous Culture	Loess and Quantile, Fourier Analysis
					[8]	ito09	800 (29%)	LL (T_{samp} 2h)	BG11 Medium, 30°C, Continuous Culture	Replicate Mean Polishing, Correlation to Sine
Microcystis aeruginosa PCC 7806	Mic7806	-	F	6360	[9]	straub11	1344 (21%)	LD (12:12, T_{samp} irreg.)	BG11 Medium, 1% air CO_2 bubbling, 22°C, Batch	LOWESS, significant difference to CT0
Anabaena sp. PCC 7120	Ana7120	•	F	6222	[10]	kushige13	78 (1.25%)	LL (T_{samp} 4h)	BG11 + N Medium, 30°C, Contiuous Culture	Replicate Mean Polishing, Correlation to Sine
Cyanothece ATCC 51142	Cyn51142	•	M	5354	[11]	stoeckel08	1445 ($\approx$ 30%)	LD (12:12, T_{samp} 4h)	ASP2 Medium, 30°C, air bubbling, Batch	LOWESS, Correlation Network
					[12]	toepel08	1424 ($\approx$ 20%)	LD (12:12)/24h, LL T_{samp} 4h)	ASP2 Medium, 30°C, Airlift Bioreactor	LOWESS, Differential Expression
					[13]	-	1400 (27%)	LD (6:6, T_{samp} 2h)	ASP2 Medium, 30°C, Airlift Bioreactor	LOWESS, Correlation Network

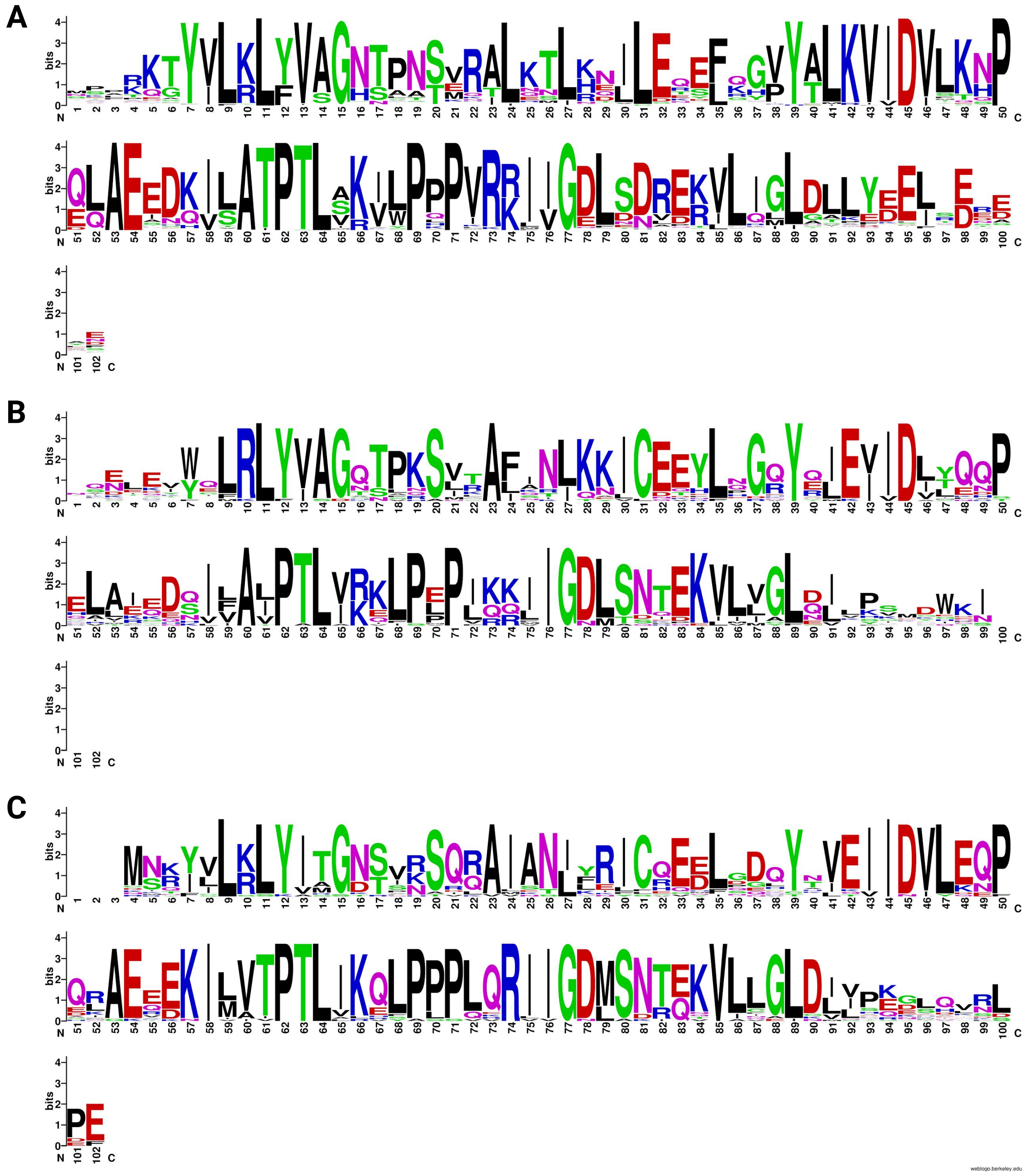

Figure S3: **WebLogos of KaiB multialignements.** All multialignments are mapped onto the sequence of KaiB from *Synechococcus elongatus* PCC 7942. (**A**) KaiB1 from *Synechocysits* sp. PCC 6803 multialignment. (**B**) KaiB2 from *Synechocysits* sp. PCC 6803 multialignment. (**C**) KaiB3 from *Synechocysits* sp. PCC 6803 multialignment.

Table S4: **Oligonucleotides used for cloning.**

Number	Primer Name	Primer Sequence	ORF
1	fw-kaiC1-Syn6714-BamHI	CTACGGATCCAACTCACCCA TCGTTAACG	D082_30580
2	rev-kaiC1-Syn6714-NotI	GAAGCGGCCGCCTACTCGAC GGTTTTATC	D082_30580
3	fw-kaiC1-Npun29133-BamHI	CTACGGATCCAGTCAAAACG AGCAAG	Npun_R2886
4	rev-kaiC1-Npun29133-NotI	CGAAGCGGCCGCTTAGGGTT CGGAAC	Npun_R2886
5	fw-kaiC1-Cy7424-BamHI	CATAGGATCCAATGAACCCA TTCCCAACG	PCC7424_0599
6	rev-kaiC1-Cy7424-NotI	CATTGCGGCCGCTTATTCAT CTAAAGTTTTATC	PCC7424_0599
7	fw-kaiC3-Cy7424-BamHI	CGAAGGATCCAATCAAGACA ACGAAC	PCC7424_3006
8	rev-kaiC3-Cy7424-NotI	CTGTGCGGCCGCCTAAGACC GTTCTTCAAAC	PCC7424_3006
9	fw-kaiC3-Mic7806-BamHI	CTACGGATCCACGCAAAATA ATCCCCTAG	IPF_2046
10	rev-kaiC3-Mic7806-NotI	GAAGCGGCCGCCTAACTACG ATCCTCA	IPF_2046
11	fw-KaiC3-PH_RS03935 BamHI (DSM 12428)	CCCGGATCCGATGCTCTTAA TTGTTGGAACTCC	Gene ID: 1443164
12	rev-KaiC3-PH_RS03935 HindIII (DSM 12428)	CGGGGAAGCTTTTACTCATA AATTTCCACCCCTC	Gene ID: 1443164
13	fw-KaiC3-OCC_RS02010 PstI (DSM 5473)	GGGCTGCAGATGAGCAGAAC GGGAATTG	Gene ID: 16548747
14	rev-KaiC3-OCC_RS02010 HindIII (DSM 5473)	GCCGGGAAGCTTTTATTCAT AAATTTCCACCC	Gene ID: 16548747

Table S5: **Diurnal core CLOGs across cyanobacterial strains** excluding *Microcystis aeruginosa* PCC 7806. In every CLOG (row) at least one gene of each of the considered datasets (columns) exhibited diurnal expression.

References

[1] V. Dvornyk, O. Vinogradova, and E. Nevo. Origin and evolution of circadian clock genes in prokaryotes. *Proceedings of the National Academy of Sciences of the United States of America*, 100(5):2495–2500, 2003.

[2] E. R. Zinser, D. Lindell, Z. I. Johnson, M. E. Futschik, C. Steglich, M. L. Coleman, M. A. Wright, T. Rector, R. Steen, N. McNulty, L. R. Thompson, and S. W. Chisholm. Choreography of the transcriptome, photophysiology, and cell cycle of a minimal photoautotroph, *Prochlorococcus*. *PLoS One*, 4(4), 2009.

[3] R. Lehmann, R. Machné, J. Georg, M. Benary, I. M. Axmann, and R. Steuer. How cyanobacteria pose new problems to old methods: challenges in microarray time series analysis. *BMC Bioinformatics*, 14:133, 2013.

[4] C. Beck, S. Hertel, A. Rediger, R. Lehmann, A. Wiegard, A. Kölsch, B. Heilmann, J. Georg, W. R. Hess, and I. M. Axmann. Daily expression pattern of protein-encoding genes and small non coding RNAs in *Synechocystis sp.* strain PCC 6803. *Applied and Environmental Microbiology*, 80(17):5195–5206, 2014.

[5] R. G. Labiosa, K. R. Arrigo, C. J. Tu, D. Bhaya, S. Bay, A. R. Grossman, and J. Shrager. Examination of diel changes in global transcript accumulation in *Synechocystis* (Cyanobacteria). *Journal of Phycology*, 42(3):622–636, 2006.

[6] K.-I. Kucho, K. Okamoto, Y. Tsuchiya, S. Nomura, M. Nango, M. Kanehisa, and M. Ishiura. Global analysis of circadian expression in the cyanobacterium *Synechocystis* sp. strain PCC 6803. *Journal of Bacteriology*, 187(6):2190–9, 2005.

[7] V. Vijayan, R. Zuzow, and E. K. O'Shea. Oscillations in supercoiling drive circadian gene expression in cyanobacteria. *Proceedings of the National Academy of Sciences of the United States of America*, 106(52):22564–8, 2009.

[8] H. Ito, M. Mutsuda, Y. Murayama, J. Tomita, N. Hosokawa, K. Terauchi, C. Sugita, M. Sugita, T. Kondo, and H. Iwasaki. Cyanobacterial daily life with Kai-based circadian and diurnal genome-wide transcriptional control in *Synechococcus elongatus*. *Proceedings of the National Academy of Sciences of the United States of America*, 106(33):14168–73, 2009.

[9] C. Straub, P. Quillardet, J. Vergalli, N. T. de Marsac, and J. F. Humbert. A day in the life of *Microcystis aeruginosa* strain PCC 7806 as revealed by a transcriptomic analysis. *PLoS One*, 6(1), 2011.

[10] H. Kushige, H. Kugenuma, M. Matsuoka, S. Ehira, M. Ohmori, and H. Iwasaki. Genome-wide and heterocyst-specific circadian gene expression in the filamentous Cyanobacterium *Anabaena* sp. strain PCC 7120. *Journal of Bacteriology*, 195(6):1276–84, 2013.

[11] J. Stöckel, E. A. Welsh, M. Liberton, R. Kunnvakkam, R. Aurora, and H. B. Pakrasi. Global transcriptomic analysis of *Cyanothece* 51142 reveals robust diurnal oscillation of central metabolic processes. *Proceedings of the National Academy of Sciences of the United States of America*, 105(16):6156–6161, 2008.

[12] J. Toepel, E. Welsh, T. C. Summerfield, H. B. Pakrasi, and L. A. Sherman. Differential transcriptional analysis of the cyanobacterium *Cyanothece* sp. strain ATCC 51142 during light-dark and continuous-light growth. *Journal of Bacteriology*, 190(11):3904–3913, 2008.

[13] J. R. Toepel, J. E. McDermott, T. C. Summerfield, and L. A. Sherman. Transcriptional analysis of the unicellular, diazotrophic cyanobacterium *Cyanothece* sp. 51142 grown under short day/night cycles. *Journal of Phycology*, 45(3):610–20, 2009.
